# Summary of the best evidence that cognitive behavioral therapy for insomnia improves sleep quality in patients with chronic insomnia

**DOI:** 10.3389/fpsyt.2025.1688561

**Published:** 2026-01-29

**Authors:** Panpan Yan, Siyu Feng, Miaomiao Ma, Bo Li, Jie Liu

**Affiliations:** 1 Huaihe Hospital of Henan University, Kaifeng, Henan, China; 2 School of Nursing and Health, Henan University, Kaifeng, Henan, China

**Keywords:** chronic insomnia, cognitive behavioral therapy for insomnia (CBT-I), evidence-based practice, non-pharmacological interventions, sleep quality

## Abstract

**Aim:**

To evaluate and summarize the best available evidence regarding the efficacy of cognitive behavioral therapy for insomnia (CBT-I) in improving sleep quality in patients with chronic insomnia and to provide structured, evidence-based recommendations for clinical nursing practice.

**Design:**

This evidence summary was conducted using the PIPOST model from the Center for Evidence-Based Nursing at Fudan University, which guided the development of structured evidence-based questions.

**Methods:**

A systematic search was performed across multiple databases and guideline repositories for literature published from inception to December 10, 2024. Literature types included clinical guidelines, best practice information sheets, expert consensuses, systematic reviews, evidence summaries, and meta-analyses. Databases searched included UpToDate, BMJ Best Practice, Joanna Briggs Institute, Guidelines International Network, National Institute for Health and Care Excellence, Embase, PubMed, Web of Science, MEDLINE, CNKI, and WanFang.

**Data sources:**

The following sources were utilized: UpToDate, BMJ Best Practice, Joanna Briggs Institute, Guidelines International Network, National Institute for Health and Care Excellence, Registered Nurses’ Association of Ontario, Scottish Intercollegiate Guidelines Network, Embase, PubMed, Sinomed, Web of Science, DynaMed, MEDLINE, CNKI, WanFang database, and Chinese Medical Journal Full-text Database. The search period was from database inception to December 10, 2024.

**Results:**

A total of 28 papers were included, comprising five guidelines, three expert consensus papers, 12 systematic reviews, and eight meta-analyses. The overall quality of the included literature was high. Forty-one pieces of best evidence were summarized across nine domains: diagnostic criteria for chronic insomnia, assessment conditions, the timing of CBT-I initiation, treatment formats, components of CBT-I, assessment metrics and tools, symptom improvement indicators, comparisons of implementers, and adverse effects.

**Conclusion:**

This study synthesized the best available evidence supporting CBT-I as an effective intervention for improving sleep quality in patients with chronic insomnia. Clinical staff should conduct comprehensive assessments before implementing CBT-I and should develop personalized treatment plans based on individual patient characteristics. The evidence supports CBT-I as a first-line treatment with minimal adverse effects compared to pharmacological interventions.

## 1 Introduction

Chronic insomnia disorder is characterized by persistent difficulty initiating or maintaining sleep despite adequate opportunity, occurring at least three times per week for a minimum of 3 months, and accompanied by impaired daytime functioning (1). This condition significantly impacts the quality of life and contributes to the progression of various systemic comorbidities, including coronary heart disease, type 2 diabetes, and obesity, through mechanisms such as inflammation, endothelial dysfunction, and metabolic dysregulation (2, 3).

This evidence summary focuses specifically on synthesizing the best available evidence for managing primary chronic insomnia in adult populations through cognitive behavioral therapy for insomnia (CBT-I). While insomnia frequently co-occurs with medical and psychiatric conditions, this review concentrates on the evidence base for CBT-I as a standalone intervention for chronic insomnia, as this represents the most robust and directly applicable body of evidence for developing standardized clinical protocols.

The primary treatment goal for insomnia is to improve sleep and reduce associated distress or dysfunction. While pharmacological interventions can offer initial relief, they are associated with risks of tolerance, dependence, and adverse effects such as falls, delirium, and daytime somnolence, particularly with long-term use (4; Colten & Altevogt, 2006). Consequently, there is a compelling need for effective non-pharmacological interventions. CBT-I, a multimodal intervention integrating cognitive therapy, behavioral strategies (e.g., sleep restriction and stimulus control), and sleep hygiene education, is recognized as the first-line treatment for chronic insomnia by leading international bodies, including the American Academy of Sleep Medicine (5).

While the efficacy of CBT-I is well-established, significant translational gaps hinder its widespread implementation in clinical practice. First, the evidence remains fragmented across numerous guidelines, systematic reviews, and meta-analyses, with occasional inconsistencies in recommendations that create confusion for clinicians. Second, there is a scarcity of structured, practical resources that synthesize this high-level evidence into actionable, step-by-step guidance—particularly for nurses and other frontline providers who are pivotal in patient assessment and non-pharmacological intervention delivery. Third, practical barriers such as a shortage of trained therapists, variable access to treatment formats, and limited institutional support impede real-world adoption. Finally, the rapid emergence of new evidence, including digital health interventions and updated culturally specific guidelines (e.g., the 2025 Chinese guidelines), necessitates a contemporary synthesis that integrates the latest developments into a coherent clinical framework.

This systematic evidence summary directly addressed these gaps. Unlike previous reviews that primarily establish efficacy, this study employed the PIPOST model—a framework specifically designed for evidence-based nursing—to translate dispersed evidence into structured, clinically actionable guidance. We aim not merely to confirm that CBT-I works but also to provide a comprehensive implementation toolkit that answers practical questions: Who should be assessed? What components should be used? How should treatment be delivered and monitored? By synthesizing the highest quality and most current evidence, including the latest guidelines, this work provides a timely resource to bridge the persistent evidence–practice divide in insomnia management.

For the purpose of this review, “sleep quality” is operationalized as a multidimensional construct encompassing both subjective measures (e.g., Pittsburgh Sleep Quality Index and Insomnia Severity Index) and objective sleep parameters, including sleep onset latency (SOL), wake after sleep onset (WASO), total sleep time (TST), and sleep efficiency (SE).

## 2 Methods

### 2.1 Problem establishment and research question

The PIPOST model (6) from the Center for Evidence-Based Nursing at Fudan University was employed to formulate structured evidence-based questions:

P (Population): adult patients with chronic insomnia disorder.

I (Intervention): multicomponent CBT-I.

P (Professional): clinicians, nurses, psychotherapists, and other healthcare providers.

O (Outcome): sleep quality indicators including SOL, WASO, TST, SE, subjective sleep quality measures, and Insomnia Severity Index (ISI).

S (Setting): medical-surgical wards, psychotherapy clinics, community settings, and home environments.

T (Type of Evidence): clinical guidelines, expert consensus statements, systematic reviews, and meta-analyses.

### 2.2 Evidence retrieval strategy

A comprehensive systematic search was conducted across multiple databases and guideline repositories from their inception to December 10, 2024. The search followed the “6S” evidence resource pyramid model, beginning with guideline databases and proceeding to systematic review and primary study databases.

English search terms included the following:

“Sleep Wake Disorder” [MeSH], “SleepWake” [Title/Abstract], “Sleep Disorders” [Title/Abstract]

“Behavioral Therapies Cognitive” [MeSH], “Cognitive Behavioral Therapies” [Title/Abstract], “Cognitive Psychotherapy” [Title/Abstract]

“Sleep hygiene education”, “stimulus control”, “sleep restriction”, “relaxation training”

“guideline” [Title/Abstract], “summary of evidence” [Title/Abstract], “Meta analysis” [Title/Abstract], “systematic review” [Title/Abstract], “consensus” [Title/Abstract]

Chinese search terms included the following:

“睡眠障碍”, “失眠”, “慢性失眠”

“认知行为治疗”, “睡眠卫生教育”, “刺激控制”, “睡眠限制”

“指南”, “专家共识”, “系统评价”, “Meta分析”

Databases and resources searched were as follows.

Guideline databases: UpToDate, BMJ Best Practice, Joanna Briggs Institute (JBI), Guidelines International Network (GIN), National Institute for Health and Care Excellence (NICE), Registered Nurses’ Association of Ontario (RNAO), and Scottish Intercollegiate Guidelines Network (SIGN).

Biomedical databases: PubMed, Embase, Web of Science, MEDLINE, CINAHL, and Cochrane Library.

Chinese databases: CNKI, WanFang Database, Chinese Biomedical Literature Database (CBM), and Chinese Medical Journal Full-text Database.

Professional organization websites: American Academy of Sleep Medicine (AASM), European Sleep Research Society (ESRS), and Chinese Sleep Research Society.

**Figure 1:**
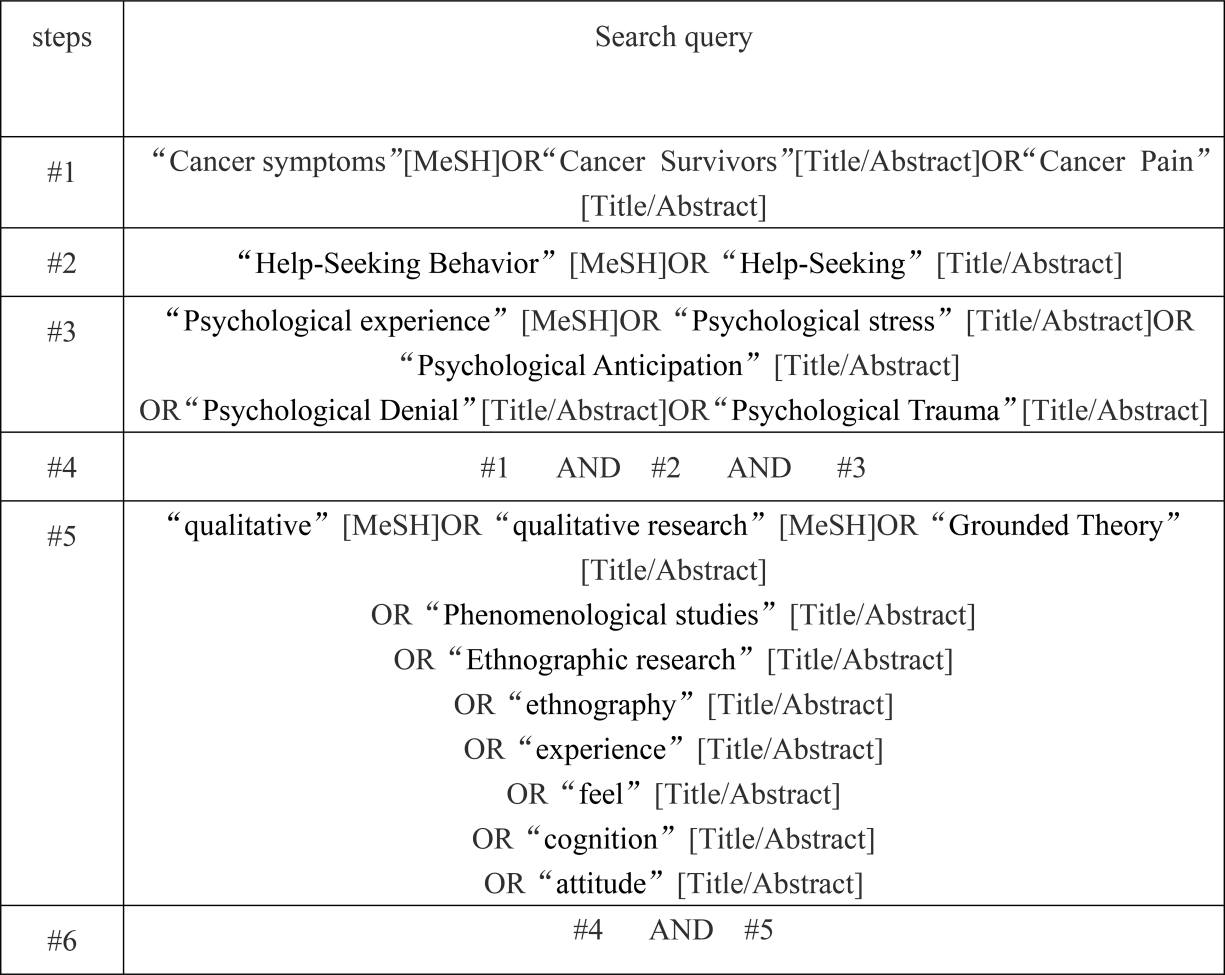
PubMed retrieval strategy.

The search strategy is shown in **Figure 1**.

### 2.3 Inclusion and exclusion criteria of evidence

Inclusion criteria:

- Study population: adults (≥18 years) diagnosed with chronic insomnia disorder.
- Intervention: multicomponent CBT-I or its specific components.
- Study types: clinical guidelines, expert consensus statements, systematic reviews, evidence summaries, and meta-analyses.
- Outcomes: sleep quality parameters, including SOL, WASO, TST, SE, and validated sleep quality scales.
- Publication languages: Chinese or English.
- Exclusion criteria:
- Translated versions of guidelines, outdated guideline versions that have been updated, or interpreted versions of guidelines.
- Duplicate publications.
- Incomplete information or inaccessible full text.
- Studies focusing exclusively on other sleep disorders (e.g., sleep apnea and parasomnias) without specific data on chronic insomnia.
- Primary research studies (randomized controlled trials and cohort studies) were not included within systematic reviews.

### 2.4 Study selection process

Two reviewers (P.Y. and S.F.), both graduate nursing students trained in evidence-based methodology, independently screened all retrieved records. The selection process followed two stages:

1. Title/abstract screening: Records were screened based on the inclusion/exclusion criteria. Clearly irrelevant studies (e.g., not about insomnia, not about CBT-I, and not a guideline/systematic review) were excluded.

2. Full-text screening: The full text of potentially eligible studies was obtained and assessed in detail.

**Figure 2:**
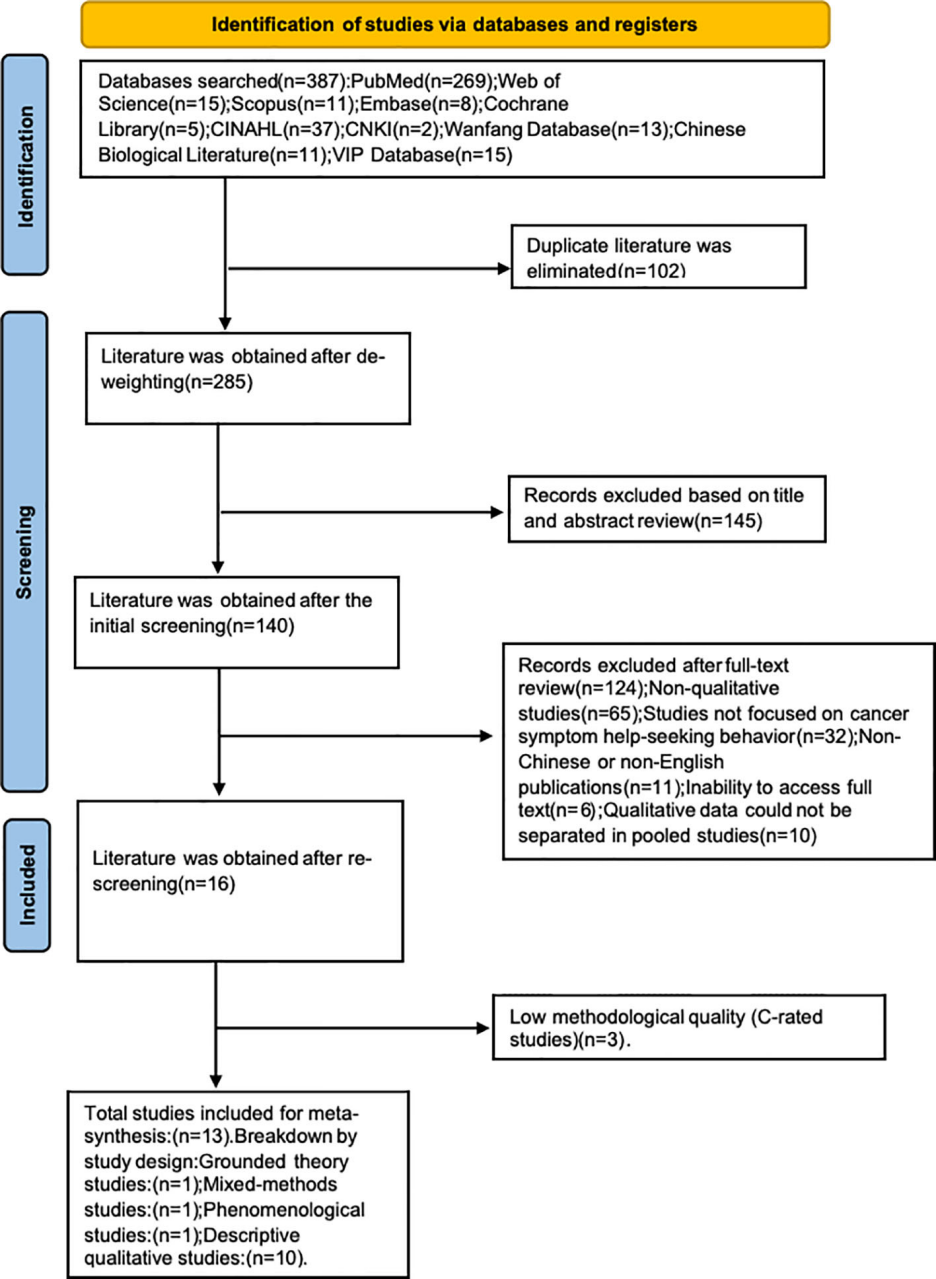
PRISMA flow diagram of study selection.

Discrepancies at any stage were resolved through discussion or by consulting a third senior researcher (B.L.). The study selection process was documented using a PRISMA 2020 flow diagram (Page et al., 2021), detailing the number of records identified, screened, excluded, and included, with reasons for exclusion at the full-text stage (see **Figure 2**).

### 2.5 Data extraction

A standardized, pilot-tested data extraction form was used. Two reviewers independently extracted data from all included studies. The extracted data included the following:

- Study characteristics: first author, publication year, country, and type of evidence (guideline/consensus/systematic review/meta-analysis).
- Population: explicit focus on adults with chronic insomnia disorder (as per study-defined criteria).
- Intervention: description of CBT-I (components, format, duration, frequency, and provider).
- Outcomes: sleep quality measures [SOL, WASO, TST, SE, Pittsburgh Sleep Quality Index (PSQI), ISI, etc.].
- Key findings and recommendations: clinical recommendations, strength of evidence, and special considerations.
- Methodological details (for quality assessment): for guidelines, development methodology; for reviews, search strategy, inclusion criteria, and risk of bias assessment method.

Any disagreements in extraction were resolved by consensus. We did not contact the original study authors for missing information, as our aim was to synthesize the published recommendations from secondary evidence sources (guidelines and systematic reviews).

### 2.6 Quality assessment

Different quality assessment tools were applied according to the literature type.

Guidelines: Appraisal of Guidelines for Research and Evaluation II (AGREE II) instrument, comprising 23 items across six domains (7). Each item was scored on a 7-point scale (1 = strongly disagree to 7 = strongly agree).

Expert consensus: JBI Critical Appraisal Checklist for Expert Opinion, containing six items evaluated as “yes”, “no”, “unclear”, or “not applicable”.

Systematic reviews and meta-analyses: JBI Critical Appraisal Checklist for Systematic Reviews and Research Syntheses, containing 11 items with the same response options.

Quality assessments were conducted independently by two reviewers, with disagreements resolved through consensus.

### 2.7 Data synthesis

Given the heterogeneity in study designs and outcomes across the included evidence, a narrative synthesis approach was employed. Evidence was organized thematically according to the PIPOST framework, with particular attention to consistency across sources and strength of recommendations.

### 2.8 Assessment of evidence certainty

Given that this review synthesized secondary evidence (guidelines, systematic reviews, and meta-analyses) rather than primary studies and employed a narrative synthesis approach, a formal assessment of evidence certainty using the GRADE framework was not conducted. Instead, a hierarchical approach was applied to evidence inclusion and synthesis: 1) prioritizing higher-level evidence (guidelines over systematic reviews, and systematic reviews over primary studies), 2) considering recency of publication, 3) evaluating methodological quality using established tools, and 4) examining consistency of findings across sources. This approach aligns with established methods for evidence summaries in nursing and implementation science.

### 2.9 Study protocol and registration

A formal protocol for this evidence summary was developed prior to commencement, but was not registered in PROSPERO or similar registries, as such registries primarily focus on systematic reviews of primary studies. The review was conducted in accordance with PRISMA guidelines, where applicable to evidence summaries, and the methodology was predetermined and consistently applied throughout the review process.

## 3 Results

### 3.1 Study selection

The systematic search identified 677 records from database searches and 28 additional records from guideline repositories and manual searches. After removal of duplicates (n = 312), 393 records underwent title and abstract screening. Of these, 265 were excluded for not meeting the inclusion criteria. The remaining 128 full-text articles were assessed for eligibility, resulting in the inclusion of 28 studies. The study selection process is detailed in the PRISMA flow diagram (**Figure 2**).

### 3.2 Overview of included evidence

The 28 included studies represented a comprehensive, high-level evidence base published between 2005 and 2025. Geographically, they originated from North America (n = 11), Europe (n = 6), Asia (n = 8), Australia (n = 2), and South America (n = 1), providing a global perspective. Temporally, 14 studies (50%) were published in or after 2020, ensuring the inclusion of contemporary evidence, including the most recent Chinese guidelines (2025). The evidence pyramid was well-represented, with clinical guidelines forming the apex, followed by expert consensuses, systematic reviews, and meta-analyses. This composition allowed for the synthesis of both authoritative recommendations and detailed efficacy data.

### 3.3 Characteristics of included studies

The 28 included studies comprised five clinical guidelines, three expert consensus statements, 12 systematic reviews, and eight meta-analyses published between 2005 and 2023. These originated from multiple regions, including North America, Europe, Asia, and Australia. Detailed characteristics are presented in **Table 1**.

**Table 1:** Characteristics of included studies (n = 28).

Included literature	Country/region	Year of publication (year)	Literature reference	Type of literature	Literature theme
Qaseem A et al. (5)	United States of America	2016	ACP	Guideline	Management of chronic insomnia disorder in adults
Riemann D et al. (8)	Europe	2023	ESRS	Guideline	Diagnosis and treatment of insomnia
Schutte-Rodin S et al. (9)	United States of America	2008	JCSM	Guideline	Assessment and management of chronic insomnia in adults
Drager et al. (10)	Brazilian	2023	ABS	Guideline	Diagnosis and treatment of insomnia in adults
Chinese Sleep Research Society (11)	China	2025	Zhonghua Yi Xue Za Zhi	Guideline	Guidelines for the Diagnosis and Treatment of Insomnia Disorder (2025 edition)
Takaesu et al. (12)	Japan	2023	PubMed	Expert consensus	Treatment strategies for insomnia
Douglas et al. (13)	Australia	2017	Elsevier	Expert consensus	Indications and performance of adult sleep studies
Palagini et al. (14)	Italy	2020	PubMed	Expert consensus	Assessment and management of insomnia
Chung et al. (15)	United States of America	2018	PubMed	Systematic review	Sleep hygiene education for the treatment of insomnia
Jun et al. (16)	United States of America	2021	Elsevier	Systematic review	Non-pharmacological interventions for insomnia in adult patients in the intensive care unit
Tamrat et al. (17)	United States of America	2013	PubMed	Systematic review	Non-pharmacological interventions to improve sleep in hospitalized patients
Seyffert et al. (18)	United States of America	2016	PubMed	Systematic review	Cognitive behavioral therapy for insomnia available on the Internet
Yu et al. (19)	China	2021	PubMed	Systematic review	Efficacy of cognitive behavioral therapy for insomnia
Zachariae et al. (20)	United States of America	2016	Elsevier	Systematic review	Efficacy of cognitive behavioral therapy for insomnia available on the Internet
Zheng et al. (21)	China	2023	PubMed	Systematic review	Effectiveness of cognitive behavioral therapy on sleep disorders in pregnant women
González-Martín et al. (22)	Spain	2023	PubMed	Systematic review	Effects of positive thought-based cognitive therapy on older adults with sleep disorders
Simon et al. (23)	Germany	2023	PubMed	Systematic review	The efficacy of cognitive behavioral therapy for insomnia
Gao et al. (24)	China	2022	Elsevier	Systematic review	Acceptability of cognitive behavioral therapy for insomnia in adults
Wang et al. (25)	Taiwan, China	2005	PubMed	Systematic review	Cognitive behavioral therapy for primary insomnia
Trauer et al. (26)	Melbourne	2015	PubMed	Systematic review	Cognitive behavioral therapy for chronic insomnia
Ho et al. (27)	Hong Kong, China	2019	Elsevier	Meta-analysis	Self-help cognitive behavioral therapy for insomnia
Forma et al. (28)	United States of America	2022	PubMed	Meta-analysis	Digital versus behavioral therapy for chronic insomnia
Ye et al. (29)	China	2015	PubMed	Meta-analysis	Internet-based cognitive behavioral therapy
Soh et al. (30)	Singapore	2020	Elsevier	Meta-analysis	Efficacy of digital cognitive behavioral therapy for insomnia
Geiger-Brown et al. (31)	United States of America	2015	Elsevier	Meta-analysis	Cognitive behavioral therapy for patients with comorbid insomnia
van der Zweerde et al. (32)	Netherlands	2019	Elsevier	Meta-analysis	Cognitive behavioral therapy for insomnia
van Straten et al. (33)	Netherlands	2018	Elsevier	Meta-analysis	Cognitive behavioral therapy for insomnia
Koffel et al. (34)	United States of America	2014	Elsevier	Meta-analysis	Cognitive Behavioral Therapy for insomnia groups

### 3.4 Quality assessment results

Guidelines: All five guidelines demonstrated generally high quality according to AGREE II criteria. Four guidelines scored ≥60% in at least five domains and were rated as “Recommended” (Category A). One guideline scored lower in applicability and editorial independence domains and was rated as “Recommended with modifications” (Category B).

Expert consensus: All three expert consensus papers met the quality criteria, with one paper having an unclear item regarding the referencing of existing literature.

Systematic reviews and meta-analyses: Among the 20 systematic reviews and meta-analyses, most items were rated as “yes” across evaluation criteria. Some studies had unclear reporting of literature inclusion criteria or methodological details, but overall quality was acceptable for inclusion.

Detailed quality assessment results are presented in **Tables 2**–**4**.

**Table 2:** Quality evaluation of the guidelines (n = 5).

Guideline	Standardized scores in various domains (%)						≧60%	≧30%	Quality evaluated
	Scope and purpose	Stakeholder involvement	Rigor of development	Clarity of presentation	Applicability	Editorial independence			
Qaseem et al. (5)	89.86%	77.78%	78.67%	98.96%	75.68%	69.76%	6	6	A
Riemann et al. (8)	69.73%	72.67%	85.65%	95.68%	68.62%	73.63%	6	6	A
Schutte-Rodin et al. (9)	87.62%	56.73%	75.65%	86.65%	78.86%	75.68%	5	6	A
Drager et al. (10)	76.32%	76.63%	75.65%	87.72%	65.42%	78.63%	6	6	A
Chinese Sleep Research Society (11)	65.41%	61.42%	56.42%	67.42%	53.25%	63.24%	4	6	B

**Table 3:** Quality evaluation of included expert consensuses (n = 3).

Expert consensuses	①	②	③	④	⑤	⑥
Takaesu et al. (12)	Yes	Yes	Yes	Yes	Yes	Yes
Douglas et al. (13)	Yes	Yes	Yes	Yes	Unclear	Yes
Palagini et al. (14)	Yes	Yes	Yes	Yes	Yes	Yes

**Table 4:** Quality evaluation of included systematic reviews (n = 20).

Systematic reviews	①	②	③	④	⑤	⑥	⑦	⑧	⑨	⑩	⑪
Chung et al. (15)	Yes	Yes	Yes	Yes	Unclear	Yes	Yes	Yes	Yes	Yes	Yes
Jun et al. (16)	Yes	Yes	Yes	Yes	Yes	Yes	Yes	Yes	Unclear	Yes	Yes
Tamrat et al. (17)	Yes	Yes	Yes	Yes	Yes	Yes	Yes	Yes	Yes	Yes	Yes
Seyffert et al. (18)	Yes	Yes	Yes	Yes	Unclear	Yes	Yes	Yes	Yes	Yes	Yes
Yu et al. (19)	Yes	Yes	Yes	Yes	Unclear	Yes	Yes	Yes	Yes	Yes	Yes
Zachariae et al. (20)	Yes	Yes	Yes	Yes	Yes	Yes	Yes	Yes	Yes	Yes	Yes
Zheng et al. (21)	Yes	Yes	Yes	Yes	Yes	Yes	Yes	Yes	Yes	Yes	Yes
González-Martín et al. (22)	Yes	Yes	Yes	Yes	Yes	Yes	Yes	Yes	Yes	Yes	Yes
Simon et al. (23)	Yes	Yes	Yes	Yes	Unclear	Yes	Yes	Yes	Yes	Yes	Yes
Gao et al. (24)	Yes	Yes	Yes	Yes	Yes	Yes	Yes	Yes	Yes	Yes	Yes
Wang et al. (25)	Yes	Yes	Yes	Yes	Unclear	Yes	Yes	Yes	Yes	Yes	Yes
Trauer et al. (26)	Yes	Yes	Yes	Yes	Yes	Yes	Yes	Yes	Yes	Yes	Yes
Ho et al. (27)	Yes	Yes	Yes	Yes	Yes	Yes	Yes	Unclear	Yes	Yes	Yes
Forma et al. (28)	Yes	Unclear	Yes	Yes	Yes	Yes	Unclear	Yes	Yes	Yes	Yes
Ye et al. (29)	Yes	Yes	Yes	Yes	Yes	Yes	Yes	Yes	Yes	Yes	Yes
Soh et al. (30)	Yes	Yes	Yes	Yes	Yes	Yes	Yes	Yes	Yes	Yes	Yes
Geiger-Brown et al. (31)	Yes	Yes	Yes	Yes	Yes	Yes	Yes	Yes	Yes	Yes	Yes
van der Zweerde et al. (32)	Yes	Yes	Yes	Yes	Yes	Yes	Yes	Yes	Yes	Yes	Yes
van Straten et al. (33)	Yes	Yes	Yes	Yes	Yes	Yes	Yes	Yes	Yes	Yes	Yes
Koffel et al. (34)	Yes	Yes	Yes	Yes	Yes	Yes	Yes	Yes	Yes	Yes	Yes

### 3.5 Evidence synthesis: consistency and convergence

The synthesis of 28 high-quality sources revealed consistent and robust evidence supporting CBT-I for chronic insomnia across all included study types and geographic regions. The five clinical guidelines unanimously recommended multicomponent CBT-I as the first-line treatment, with particularly strong and updated endorsement in the most recent 2025 Chinese guidelines. The 12 systematic reviews and eight meta-analyses provided convergent evidence for CBT-I’s efficacy across diverse delivery formats (individual, group, and digital) and patient populations.

Key convergent findings across all evidence types included the following:

1. Superior risk–benefit profile compared to pharmacological interventions.
2. Effectiveness across multiple delivery formats, with face-to-face individual therapy as the gold standard and digital formats offering comparable efficacy with greater accessibility.
3. Critical importance of multicomponent approaches over the use of single components (e.g., sleep hygiene alone).
4. Feasibility of implementation by trained non-specialists, including nurses, increasing the potential for wider dissemination.

Crucially, no major contradictions were found between older and newer evidence. Recent guidelines (e.g., 8, 11) primarily refined and strengthened earlier recommendations, particularly regarding the role of digital delivery and safety considerations for specific populations (e.g., older adults).

### 3.6 Summary of best evidence and clinical pathway

Thematic synthesis yielded 41 discrete pieces of best evidence, organized into nine clinically actionable domains (**Table 5**). The evidence demonstrated high consistency, with any variations (e.g., in recommendations for specific populations) reflecting appropriate clinical nuance rather than fundamental disagreement about CBT-I’s core value.

**Table 5:** Summary of best evidence for CBT-I to improve sleep quality in chronic insomnia.

Category	Content of evidence	Level	Recommendation level
Diagnostic criteria	1. Sleep latency of more than 30 minutes, remaining awake for more than 30 minutes after falling asleep, sleep efficiency of less than 85%, or total sleep time of less than 6–6.5 hours, with complaints of at least 3 nights per week for at least 3 months (25, 28, 29).	A	1
Conditions of assessment	2. The assessment of insomnia involves evaluating the type, frequency, and duration of nighttime sleep, as well as the sleep environment. In addition, the patient’s daytime lifestyle, such as type of work, social activities, diet, and exercise, needs to be assessed in order to rule out its interfering factors contributing to insomnia (25; Steinmetz L et al., 2023; 24).	c	1
	3. Treat patients with CBT-I based not only on recommended suggestions but also on clinical experience, previous patient responses, and patient preferences and potential adverse effects (15, 19, 28).	a	1
	4. It is recommended that the health risks of insomnia and the availability of psycho-behavioral treatments be made available to general practitioners (15).	a	1
	5. Residents should develop standardized inpatient sleep protocols for hospitalized patients to minimize sleep disruption, such as not performing vital sign monitoring, medications, and other therapeutic measures during sleep time if not necessary (17).	c	1
Therapeutic target	6. The goal of treatment for insomnia is to improve sleep and reduce the pain or dysfunction caused by the disorder (Ree M et al., 2017; Cartwright R et al., 2014).	a	1
Timing of activation	7. After a thorough evaluation of the patient, patients diagnosed with insomnia can start CBT-I treatment while treating their underlying disease (Chung KF et al., 2018; Tamrat R et al., 2014).	c	1
Forms of treatment	8. Including face-to-face individual therapy, face-to-face group therapy, telephone-based follow-up therapy, web-based modular therapy, and self-help book therapy.	c	1
	9. Of which face-to-face individual therapy is the most recommended, and Internet-assisted CBT-I should be considered as the first choice when face-to-face CBT-I is limited by factors such as time, location, and cost of treatment (27).		
	10. Compared with Internet-delivered CBT-I, face-to-face CBT-I was more effective in decreasing SOL and WASO severity, but there was no significant difference in post-treatment efficacy between these two modalities when compared with face-to-face CBT-I. There was no significant difference in efficacy after treatment between these two modalities, and Internet-assisted CBT-I is less costly (18–21, 23, 24, 28–30, 34).		
	11. Individual CBT-I may be most effective in improving insomnia severity, and the CBT-I group may be most effective in improving WASO. Digitally assisted CBT-I may be most effective in improving sleep efficiency, SOL, and TST (27, 28).		
	12. Guided self-help and non-guided self-help CBT-I are less acceptable than individual and group CBT-I.		
	13. Self-help treatment is effective but not for everyone (22, 27).	a	5
	14. If cognitive behavioral therapy for insomnia (CBT-I) alone is unsuccessful, additional pharmacotherapy is recommended for patients with chronic insomnia disorders, with only short-acting benzodiazepines recommended, and extended-release melatonin is more recommended for older adults. Patients with insomnia that does not resolve within 7–10 days of combination therapy should be further evaluated (12).	a	5
	15. There is no evidence that CBT-I combined with medication is superior to CBT-I alone in the treatment of insomnia (9, 15, 19).	a	5
Components of CBT-I			
Sleep hygiene education (SH)	16. Sleep hygiene education is recommended as first-line treatment when benzodiazepine hypnotics are discontinued (35). However, the single use of sleep hygiene education to improve insomnia symptoms is not recommended (9, 15, 19).	a	5
	17. It is recommended that those who use benzodiazepines for long-term treatment of insomnia disorders should taper off the medication when it is discontinued and try to use sleep hygiene education early on to alleviate adverse symptoms.		
	18. Poor sleep hygiene is a prerequisite for the use of sleep hygiene education, which can be considered the first step in the treatment of insomnia when CBT-I is not available for various reasons (15).	a	1
Stimulus control (SC)	19. Low-quality evidence suggests that stimulus control improves sleep latency and total sleep time in the general population and has been shown to be superior to other treatment modalities such as progressive relaxation, imagery training, and paradoxical intentions, but it is not recommended for use in older people (10).	a	5
Cognitive therapy (CT)	20. CT is superior to any single-component treatment and helps to break the vicious cycle of sleep disorders and reduce the risk of self-medication. Limited evidence suggests that positive thinking can be introduced to correct poor short- and long-term sleep quality (15, 22, 32, 33).	a	1
Sleep restriction (SRT)	21. Some potential adverse effects, such as fatigue, excessive daytime sleepiness, and difficulty concentrating, may occur early in treatment. Therefore, it is not recommended for the following groups: people in high-risk occupations (drivers and heavy machinery operators) and people with excessive daytime sleepiness. Studies have shown that restricting the duration of sleep can increase the risk of falls in elderly patients (10, 15, 18; Yu Zhang et al., 2021).	a	1
Relaxation training (RE)	22. Relaxation training has been used to reduce anxiety, pain, blood pressure, and heart rate, among others, and its application is recommended to improve the quality of sleep in patients. However, as a standalone treatment, relaxation training is not as good as the other components of the practice (17).	c	1
	23. Relaxation training requires more training sessions and longer intervals than sleep restriction (17).	c	1
	24. Phototherapy and exercise interventions can be used as adjunctive therapies to CBT-I (22).	a	1
Treatment cycle	25. An average of 8 weeks with 4 to 8 sessions per week, i.e., 4 to 8 individual or group sessions per week or every two weeks, lasting an average of 60 to 90 minutes. The lower the frequency and the shorter the duration of the intervention, the fewer techniques it contains (22, 31, 33).	c	1
	26. It is recommended that when CBT-I is performed face-to-face, a minimum of 4 sessions is the optimal therapeutic dose, and 5 or more sessions are more effective (31, 33).	a	1
	27. The therapeutic effect of CBT-I is stable, but intensified sessions are required over time to restore the initial therapeutic effect (22, 33).	c	1
	28. Due to the high recurrence rate of insomnia, sleep indicators should be reassessed every 6 months after the end of treatment to adjust the treatment program in a timely manner (32).	c	1
	29. Increasing the number of follow-up telephone counseling visits improves treatment adherence for CBT-I (25).	a	1
Adverse reaction	30. Due to the non-invasive nature of CBT-I, no or very mild adverse effects have been reported. Therefore, CBT-I provides better overall value than drug therapy (12–17).	a	5
Indicators for assessing sleep outcomes	31. Primary outcome indicators: sleep onset latency (SOL) and wake after sleep onset (WASO).	a	5
	Secondary outcome indicators: total sleep time (TST), sleep efficiency (SE), sleep quality, and insomnia severity index (ISI) (14–16).		
Sleep Outcome Assessment Tool	32. Questionnaires (Epworth Sleepiness Scale), Pittsburgh Sleep Quality Index (PSQI), Insomnia Severity Inventory (ISI), Epworth Sleepiness Scale (ESS), and Activity Recorder are recommended for assessing outcomes and guiding insomnia (10, 13, 26).	a	5
	33. It is recommended to begin treatment using a sleep diary to record insomnia symptoms for at least 1 to 2 weeks to monitor the dynamics of insomnia and to complete the sleep diary for an additional 1 to 2 weeks post-treatment and at follow-up visits (26).	a	1
	34. Polysomnography (PSG) is not recommended for routine use in the evaluation of insomnia but may be used to rule out other subtypes of sleep disorders (lifelong insomnia, sleep state delusions, and insomnia due to poor sleep habits) or when there is no response to medication (Jun J et al., 2021).	c	2
	35. Activity loggers are not recommended as a diagnostic tool for insomnia; they must be used in conjunction with other clinical information and patient history, and they are less sensitive to changes in insomnia following CBT-I than subjective measures such as sleep diaries (Drager, L. F et al., 2023).	a	5
	36. If circadian rhythm disruption is suspected, wearing an activity recorder is recommended to assess sleep duration (Gao Y et al., 2022).	c	1
	37. The Insomnia Severity Inventory (ISI) scores range from 0 to 28, with 8–14 indicating subclinical insomnia, 15–21 indicating moderate insomnia, and 22–28 indicating severe insomnia. When assessing insomnia disorders in community settings, a change score of 8.4 is recommended as a marker of moderate improvement (13, 26)	c	1
	38. Sleep efficiency (SE) <80% is the cutoff value that distinguishes insomniacs from good sleepers (9–11).	a	1
Symptom improvement indicators	39. (1) Reduction of the main target symptom (sleep latency or time awake after sleep onset) by more than 50%.	a	1
	(2) Proportion of patients whose sleep efficiency changes from dysfunctional to normal levels (>80%–85%).		
	(3) Decrease in hypnotic drug use (19–22).		
Selection of CBT-I implementers	40. There is still a shortage of trained therapists, and evidence suggests that treatment delivered by professionals (e.g., psychologists) has better outcome indicators than trainees. However, the therapeutic effects of treatment delivered by trainees are very similar and equally effective as those achieved by trained mental health professionals (18, 25, 31).	c	1
	41. General psychotherapy is usually unable to alleviate insomnia, so clinicians should receive specialized training in CBT-I theory and implementation, which may include attending specialized CBT-I seminars and online CBT-I courses (14).	a	1

The synthesized evidence clearly delineates a three-stage clinical pathway for implementation:

1. Comprehensive assessment: utilizing validated tools (e.g., sleep diaries and ISI) to establish diagnosis and baseline.
2. Personalized treatment planning: selecting appropriate CBT-I components and delivery formats based on individual patient characteristics, preferences, and comorbidities.
3. Systematic monitoring and follow-up: tracking outcomes and adverse effects to adjust treatment and ensure long-term benefit.

The following section provides a narrative summary of the key findings within each domain, with the complete evidence detailed in **Table 5**.

### 3.7 Key findings by domain

Diagnostic criteria: Chronic insomnia is diagnosed when sleep latency exceeds 30 minutes, wake after sleep onset exceeds 30 minutes, sleep efficiency is below 85%, or total sleep time is less than 6–6.5 hours, occurring at least three nights per week for 3 months or longer.

Assessment conditions: A comprehensive assessment should evaluate sleep patterns, the sleep environment, daytime functioning, lifestyle factors, and potential contributing medical or psychiatric conditions prior to initiating CBT-I.

Treatment formats: While face-to-face individual CBT-I is most strongly recommended, digital/telehealth formats demonstrate comparable efficacy with the advantages of increased accessibility and lower cost, making them a viable first-line alternative when barriers exist.

CBT-I components: Multicomponent CBT-I (typically including sleep restriction, stimulus control, cognitive therapy, and sleep hygiene education) is consistently more effective than any single-component approach used in isolation.

Treatment duration: An effective course typically involves four to eight sessions over 6–8 weeks, with at least four sessions considered a minimum for a therapeutic effect.

Assessment tools: Sleep diaries are recommended for baseline assessment and ongoing monitoring, complemented by validated questionnaires (e.g., PSQI and ISI). Polysomnography is not routinely recommended unless other sleep disorders are suspected.

Implementation: CBT-I can be effectively delivered by trained clinicians or nurses or through guided self-help formats. While specialized therapist training enhances outcomes, evidence supports the effectiveness of trained non-specialists, which is crucial for scalability.

Safety: CBT-I has a minimal adverse effect profile, primarily limited to transient fatigue or sleepiness during initial treatment phases. This favorable safety profile stands in stark contrast to the risks associated with long-term pharmacological treatment.

## 4 Discussion

This evidence synthesis integrates contemporary guidance on CBT-I for chronic insomnia. A key strength is the inclusion of the latest Guidelines for the Diagnosis and Treatment of Insomnia Disorder (2025 Edition) from the Chinese Sleep Research Society, which ensures that the recommendations reflect recent evidence and are relevant for clinical practice, particularly within Chinese healthcare contexts. The following sections discuss key clinical implications derived from the synthesized evidence.

### 4.1 Comprehensive assessment and individualized treatment planning

Consistent evidence from international and recent national guidelines supports multicomponent CBT-I as the first-line treatment for chronic insomnia (5, 8, 11). However, effective implementation requires a thorough patient assessment to identify the individual biological, psychological, and social factors influencing sleep. Clinical staff should conduct comprehensive evaluations encompassing sleep history and patterns (e.g., via sleep diaries), medical and psychiatric comorbidities, current medication and substance use, lifestyle and occupational factors, and patient preferences and goals before initiating CBT-I. This aligns with the 2025 Chinese guidelines’ emphasis on a holistic assessment to inform personalized management (11).

For patients with comorbid conditions, CBT-I components should be strategically tailored. For instance, treatment may emphasize cognitive restructuring to address sleep-related anxiety in depression or integrate relaxation techniques more prominently for pain-related insomnia. Importantly, the presence of comorbidities does not diminish the efficacy of CBT-I but necessitates adjustments in treatment focus, pacing, and potential interdisciplinary collaboration (Sweetman et al., 2017). Practical application includes the following: for patients with depression, early introduction of cognitive therapy to counter hopelessness about sleep; for those with chronic pain, pairing sleep scheduling with paced activity and relaxation; and for older adults, modifying sleep restriction protocols to minimize daytime sleepiness and fall risk.

Clinical implication for assessment: To operationalize this evidence, clinical units should develop a standardized insomnia assessment toolkit. This toolkit could include 1) a one-page sleep history form capturing the criteria in Evidence #1-2, 2) the ISI for baseline severity, 3) a 1-week sleep diary template, and 4) a checklist for common comorbidities and medications affecting sleep. Nurses can be trained to administer this initial assessment, streamlining the referral process to CBT-I providers and ensuring treatment begins with a comprehensive understanding of the patient’s sleep problem.

### 4.2 Flexible treatment delivery and monitoring

Evidence supports multiple CBT-I delivery formats with comparable efficacy. While face-to-face individual therapy remains the gold standard, digital/telehealth options provide viable alternatives when access, cost, and convenience are concerns. Clinical staff should consider patient preference, technological literacy, and support needs when selecting a delivery format. During treatment, regular monitoring of sleep parameters and daytime functioning allows for timely adjustment of intervention components. For example, if sleep restriction initially increases daytime sleepiness, a slight modification of time in bed may be warranted while maintaining therapeutic principles.

Tailoring CBT-I for Comorbid Insomnia (Practical Application): The evidence supports CBT-I for patients with comorbid conditions, but its implementation requires adaptation. Clinicians should use a “CBT-I Plus” approach.

For insomnia with depression/anxiety: Initiate treatment with cognitive therapy to directly address the negative, ruminative thoughts (“I’ll never sleep”) that fuel both mood and sleep disturbances. Behavioral experiments (e.g., testing the belief that “poor sleep will ruin my next day”) can be powerful.

For insomnia with chronic pain: Integrate relaxation training (e.g., progressive muscle relaxation) at the outset to address hyperarousal and pain-related tension. Modify sleep restriction cautiously to avoid exacerbating pain through fatigue.

For insomnia in older adults: Prioritize stimulus control and cognitive therapy over strict sleep restriction. Educate on fall prevention (e.g., using nightlights and clear pathways) when implementing stimulus control. Consider slightly higher sleep efficiency targets (e.g., >75% instead of >85%) initially.

For all comorbidities: Collaborate with the treating specialist (e.g., psychiatrist and pain specialist) to align treatment goals and messaging. The evidence is clear: treating insomnia improves comorbid symptoms, and treating the comorbidity does not preclude successful insomnia therapy.

### 4.3 Evidence-based use of assessment tools

Validated instruments enhance assessment accuracy and treatment monitoring. Sleep diaries should be utilized for at least 1–2 weeks pre-treatment and during therapy to capture sleep patterns and variability. The ISI provides a reliable measure of treatment response, with a reduction of ≥8 points indicating clinically meaningful improvement. Activity monitors may supplement assessment but should not replace subjective measures or clinical evaluation. Polysomnography is reserved for cases where other sleep disorders are suspected or when initial treatment proves ineffective.

Clinical implication for monitoring: The sleep diary is not just an assessment tool but a core interventional component of CBT-I. Clinicians should use it proactively: review diaries with patients to identify patterns, collaboratively set goals based on the data (e.g., “Let’s try to reduce your time in bed to match your actual sleep time this week”), and use the diary’s objective record to challenge unhelpful beliefs about sleep (e.g., “You believed you slept only 4 hours, but your diary shows 6.5 hours”). This transforms assessment into a therapeutic process.

### 4.4 From evidence to practice: a roadmap for nursing implementation and training

The synthesized evidence not only establishes CBT-I’s efficacy but also provides a clear mandate and roadmap for nurses to assume a leading role in insomnia management. Nurses are uniquely positioned across the care continuum—from primary care and hospitals to community settings—to implement evidence-based sleep interventions.

Step 1: Foundation—Education and training healthcare systems must invest in building nursing capacity in behavioral sleep medicine. This includes the following:

- Integrating foundational content on sleep health, insomnia assessment, and CBT-I principles into nursing education curricula.
- Developing specialized, competency-based training programs for practicing nurses, which may range from short certifications to supervised clinical practicums.
- Establishing train-the-trainer models to create local champions who can sustain and spread knowledge within their institutions.

Step 2: Integration—Building structures for practice nurses can leverage the evidence to advocate for and build supportive practice structures:

- Implement standardized screening: Incorporate brief, validated tools like the ISI into routine nursing assessments across settings.
- Develop clinical protocols: Create nurse-led pathways for insomnia, starting with sleep hygiene education and stepped care, supported by institutional sleep protocols to minimize iatrogenic disruption in hospitals (Evidence #5).
- Formalize nurse-led services: Advocate for policies that recognize, credential, and reimburse nurse-delivered behavioral sleep interventions, legitimizing this expanded scope of practice.

Step 3: Delivery—Executing and adapting care with training and structures in place; nurses can directly deliver and coordinate CBT-I:

- Lead core components: Deliver psychoeducation, sleep hygiene, stimulus control, and relaxation training while collaborating with psychologists or physicians for advanced cognitive therapy or sleep restriction when needed.
- Embrace digital health: Utilize telehealth platforms to increase access, provide follow-up, and support adherence, particularly for patients in remote areas or with mobility issues.
- Tailor to context and comorbidity: Apply the principles for comorbid populations (Section 4.2) and adapt delivery to specific settings (e.g., brief interventions in primary care and more structured programs in specialty clinics).

Step 4: Evaluation—Ensuring quality and impact sustained implementation requires ongoing evaluation:

- Monitor patient outcomes using the sleep diaries and validated scales recommended in the evidence.
- Track program metrics such as reach, adherence, nurse competency, and patient satisfaction.
- Use data for continuous improvement of protocols and training, ensuring interventions remain effective, efficient, and patient-centered.

This roadmap translates the 41 pieces of best evidence into a concrete, phased action plan. By progressing from education to evaluation, nursing can systematically close the evidence–practice gap in insomnia care, improving access to first-line treatment and patient outcomes.

### 4.5 Safety and integration with pharmacotherapy

CBT-I demonstrates an excellent safety profile with minimal adverse effects, primarily transient fatigue or sleepiness during initial treatment—a finding consistently affirmed across international and the updated Chinese guidelines. When considering integration with pharmacotherapy, the evidence prioritizes CBT-I as the foundational treatment, using medications only adjunctively for short-term relief if needed. The 2025 Chinese guidelines reinforce this stance, emphasizing that CBT-I should be offered as first-line treatment and that hypnotic medications are not recommended for long-term use. For patients transitioning from long-term hypnotic use, CBT-I can facilitate a safer taper while managing withdrawal symptoms. Collectively, the evidence indicates that CBT-I provides better long-term value and sustained benefits beyond treatment cessation compared to medication alone, with the added advantage of avoiding risks associated with dependence and tolerance.

## 5 Limitations

This evidence summary has several methodological and practical limitations that should be considered when interpreting and applying its findings.

### 5.1 Methodological limitations

#### 5.1.1 Language and database bias

The search was restricted to Chinese and English literature, potentially omitting relevant evidence published in other languages. Although major international databases were searched, some regional or gray literature may not have been captured.

#### 5.1.2 Synthesis constraints

The inherent heterogeneity in study designs, outcomes, populations, and reporting formats across the included guidelines and systematic reviews precluded a quantitative meta-analysis. Consequently, a formal assessment of the overall certainty of evidence using the GRADE framework was not conducted.

#### 5.1.3 Dependence on secondary sources

As a synthesis of secondary evidence (guidelines and systematic reviews), our conclusions are contingent upon the methodological rigor, scope, and potential biases of the original publications. We did not re-appraise the risk of bias in the primary studies included within each systematic review.

#### 5.1.4 Temporal limitation

The field of digital therapeutics and behavioral sleep medicine is rapidly evolving. Our search cutoff date (April 2024) may not capture the latest evidence, particularly for novel digital delivery formats and applications in emerging populations.

### 5.2 Practical and generalizability limitations

#### 5.2.1 Evidence–practice gap

While the evidence robustly supports CBT-I’s efficacy, most of the evidence derives from controlled research settings. The synthesis may not fully capture the practical barriers to implementation (e.g., time constraints, reimbursement issues, and staff training needs) encountered in routine clinical practice, especially in resource-limited settings.

#### 5.2.2 Cultural and contextual factors

Although we included the culturally informed 2025 Chinese guidelines, the majority of the evidence originates from Western healthcare contexts. The direct applicability of these recommendations and the necessary cultural adaptations for diverse global populations require further consideration and local validation.

Despite these limitations, this systematic synthesis provides a comprehensive and contemporary foundation for implementing CBT-I. The structured presentation of best evidence across nine clinical domains offers a valuable roadmap. The findings should be applied judiciously, integrating clinical expertise with consideration of local resources, patient preferences, and cultural context, while engaging in ongoing evaluation of implementation outcomes.

## 6 Conclusion and implications for nursing practice

This systematic evidence summary synthesizes high-quality, contemporary guidance supporting CBT-I as an effective, safe first-line intervention for chronic insomnia. Beyond establishing efficacy, this review provides a structured framework for clinical implementation through the PIPOST model, addressing the critical gap between evidence and practice.

For nursing practice, the evidence supports several key actions: 1) systematic assessment of insomnia in diverse patient populations using validated tools; 2) advocacy for CBT-I as the preferred initial treatment over pharmacological interventions; 3) development of nurse-led insomnia management programs, particularly in settings with limited access to sleep specialists; 4) tailored implementation of CBT-I components based on individual patient characteristics and comorbidities; and 5) integration of digital health solutions to enhance accessibility and adherence.

Future implementation should focus on developing standardized nursing protocols for insomnia assessment and management, creating training programs to build nursing capacity in behavioral sleep medicine, establishing systems for monitoring treatment response and safety, and conducting implementation research to identify optimal strategies for integrating CBT-I into diverse care models. By bridging the evidence–practice gap, nurses can play a pivotal role in improving sleep health outcomes for individuals with chronic insomnia.

## Data Availability

The original contributions presented in the study are included in the article/supplementary material. Further inquiries can be directed to the corresponding author.
